# Highlights from the 2013 WIN Symposium: personalised cancer therapy from innovation to implementation

**DOI:** 10.3332/ecancer.2013.344

**Published:** 2013-08-29

**Authors:** Richard L Schilsky

**Affiliations:** American Society of Clinical Oncology, Alexandria, VA, 22314 USA

**Keywords:** biomarkers, personalised medicine, targeted therapy, genomics, proteomics

## Abstract

The Worldwide Innovative Networking (WIN) consortium is a global alliance of academic and industrial cancer researchers, clinicians, and cancer advocacy groups set up to promote innovations in personalised cancer therapy and to accelerate the translation of research in this discipline into the oncology clinic. One of its most important initiatives is the WIN symposia, which have been held in Paris each summer since 2009. The fifth WIN symposium, which was held 10–12 July 2013, took as its overall theme ‘Personalised Cancer Therapy: From Innovation to Implementation’.

Over 400 delegates, including a good number of representatives of patient groups as well as leading academic, industrial, and clinical scientists; students; and post-docs attended this symposium. Its scientific programme featured thirty presentations divided into four main plenary sessions, and there was also a wide-ranging poster session that encompassed all the topics covered in the plenaries.

The programme structure followed the path of drug discovery, in that the first session covered assay development for personalised cancer medicine; the second, applications of genomics in oncology; the third, clinical development; and the fourth, the impact of personalised medicine on cancer care.

The first session at the 2013 WIN symposium covered genomic and proteomic technologies for the development and use of assays, biomarkers, and molecular diagnostics; it was chaired by Levi Garraway of the Dana-Farber Cancer Institute, Boston, United States. With the increasing pace of discovery of oncogenic genetic aberrations and their associated biomarkers, basic scientists and clinical oncologists now need to understand and work with complex molecular datasets. Therefore, this session began with a number of presentations describing new developments in genomics, proteomics and—crucially—bioinformatics that support oncology research and clinical decision making.

It took over ten years and cost billions of dollars to sequence the first human genome. That project ended in 2003, and since then genome sequencing has become almost routine. Clinicians, including Arul Chinnaiyan of the University of Michigan at Ann Arbor, United States, described how they can now use next-generation sequencing to build up a complete picture of genetic aberrations in a patient’s tumour within a clinically relevant time frame. At Ann Arbor, results are presented to a board of experts to decide which, if any, prescription or investigational drugs can be given. Chinnaiyan cited a case of acute paediatric leukaemia that was, unusually, found to bear a mutation that could be treated with imatinib.

Genomics is not the only high-throughput molecular technology to have important applications in cancer therapy. Equally useful results can be derived with proteomics applied to oncology. Christoph Borchers from the University of Victoria in Canada started his talk by highlighting *Nature Methods*’ selection of ‘targeted proteome analysis’ as its Method of the Year for 2012. He described methods that have been developed to improve the stability of tumour samples and the reliability and robustness of high-throughput protein analysis. His group is now developing methods of extracting and quantifying protein biomarkers from blood samples, which are self-evidently much easier to obtain than tumour biopsies. Emanuel Petricoin of George Mason University, Manassas, Virginia, United States, moved beyond the proteome to discuss cancer as a disease of defective protein networks and described the use of ‘pathway mapping’ to sub-divide triple-negative breast tumours based on which proteins—particularly which protein kinases—appear to be active. Pilot studies suggest that drugs selected on this basis are likely to prove effective.

The talks presented in the second and third sessions concerned developments in the clinical application of genomics and proteomics, starting with generic applications and moving on to specific cancer types. Lisa McShane of the US National Cancer Institute discussed the important issue of the clinical trial designs for biomarkers and biomarker-based therapeutics. Several novel trial designs have been developed, including so-called umbrella trials in which each patient is screened for biomarkers and assigned to the most appropriate trial arm based on the results of the screen. These present logistical challenges, not least because of the large number of patients that must be screened, and international cooperation is needed to make sure that the maximum number of patients can benefit from such trials without over-duplication.

Continuing the progression ‘from bench to bedside’, Scott Ramsey of Fred Hutchinson Cancer Research Center, Seattle, Washington, United States, described how genomics and proteomics are starting to influence every point in cancer management, from risk assessment to predicting drug toxicity. He suggested that the greatest challenge to its wider introduction is likely to be its high cost, particularly outside the United States, and suggested that innovative methods of sharing cost between developers and providers need to be established to ensure that suitable patients can access novel drugs. The next presenter, Rui Manuel Reis from Barretos Cancer Hospital in San Paolo, described the challenges involved in and opportunities for setting up personalised cancer medicine in Brazil, which, although rapidly developing, is still a middle-income country.

The next section of talks described the use of molecular profiling in several common tumour types: colorectal cancer; breast cancer, with presentations from China and the United Kingdom; and melanoma. In the last of these, Alexander Eggermont (Institut Gustave Roussy, Villejuif, France) explained how a combination of molecular profiling and the development of immunotherapies and ‘small molecule’ drugs to target specific mutations was beginning to transform the prospects for patients with this hard to treat tumour type.

Despite many promising results, particularly in small-scale trials, the extent to which patients can be matched to drug regimens by their molecular profiles can improve the outcome for all patients. The WIN consortium has designed a large clinical trial, named WINTHER, to assess this, and interim results from this trial were presented by Jean-Charles Soria from the Institut Gustave Roussy, Villejuif, France. The main unique feature of this study, which is still recruiting patients, is that it includes patients with no DNA aberration that can be matched to a registered or investigational targeted therapy: that is, the majority of patients. Tumour and matched normal DNA from patients with a variety of metastatic cancers is sequenced; patients with so-called actionable mutations are treated with drugs that are appropriate for those mutations, while others are treated either with standard care or with drugs chosen using an algorithm based on the RNA profiles of their tumours.

The final section of talks discussed how these new approaches are already having an impact on the care of cancer patients, even in tumour types with a historically poor prognosis. Daniel Catenacci of the University of Chicago, Illinois, United States, described how he is using biomarkers to select appropriate therapies for patients with advanced cancer of the gastro-oesophagus, and David Chang of the University of Glasgow, United Kingdom, explained how molecular profiling is being used to identify the rare patients with pancreatic cancer who will respond well to gemcitabine. Finally, Drew Pardoll of Johns Hopkins University, Baltimore, Maryland, United States, returned to the topic of immunotherapy, suggesting that combined with molecular profiling, it should be seen as a ‘fifth pillar’ of cancer treatment, alongside and equally important as surgery, radiotherapy, chemotherapy, and targeted small molecule drugs.

The findings presented in the symposium were summed up by Richard Schilsky, chief medical officer of the American Society of Clinical Oncology (ASCO) and chairman of WIN’s scientific advisory board. Schilsky started his concluding remarks by summarising the essential schema that have emerged for personalised cancer medicine. Molecular profiling is essentially replacing the original, simple classification of tumours by site and histology. DNA taken from a tumour biopsy is analysed along with normal DNA from the same patient to obtain a set of somatic mutations that are unique to that patient’s tumour. The promise of personalised medicine is that it will eventually be possible to select a therapy or, more likely, a combination of therapies that is as unique to that patient as the molecular profile. And it should be remembered that cancer is a dynamic disease, and both the mutation set and the optimum treatment are subject to change during its course.

The era of targeted therapy, however, has brought with it a number of challenges for researchers and clinicians. With the fragmentation of tumour types into more sub-types with different molecular profiles, there is a pressing need for drugs that have been optimised to treat them. And drugs that have been designed to match as perfectly as possible a distinct molecular profile will, self-evidently, reach a small group of patients and will not have the same marketing opportunities as less targeted drugs. Clinical trials are becoming more complex and expensive with the need to recruit patients with specific tumour profiles in sufficient numbers. There is much that we still do not know, including the best way to test and optimise the dosing schedule and sequence for the multiple drugs that are likely to be prescribed for most cancer patients.

Schilsky next cited three of the problems that have been raised by the increased use of molecular profiling and the development of individually targeted therapies and presented some possible solutions. First, he addressed some of the difficulties involved in collecting and testing tumour tissue. As yet, there are no established and validated guidelines for how such tissue should be obtained, handled, tested, and stored. Several independent platforms for the molecular and genetic profiling of tissue have become established, but there is still little guidance even on how positive and negative test results should be defined. ASCO has collaborated with other US-based professional bodies, including the College of American Pathologists, to produce several sets of guidelines for the handling and testing of tumour tissue in some specific cases. One example is the ASCO-CAP guidelines for HER2 testing in breast cancer, which aim to improve the consistency and accuracy of this. These set out and recommend an algorithm for FISH testing of HER2 that includes strict definitions of positive, negative, and equivocal test results. Similar guidelines have been set out for determining EGFR mutation and ALK rearrangement status in lung tumours. It is necessary and increasingly possible to assist physicians in the genetic analysis of tumours; the website www.mycancergenome.org provides a tool for matching mutations to recommended treatments that is accessible enough for busy clinicians to use.

Currently, however, the biomarkers and diagnostic tests that are available—let alone validated—represent only a tiny fraction of what will be possible when ‘cancer genomes’ have been fully elucidated. Schilsky chose the increasing complexity of molecular diagnosis as the second problem to be presented. Eventually, it should be possible to determine an integrated genomic profile from each patient and to use a published algorithm to decide on the most appropriate treatment plan. This will need an enormous amount of work to determine and validate appropriate biomarkers and tests for each possible aberration. And it will be important to roll new and well-validated tests out as widely as possible. In France, the Institut National de Cancer has established 28 regional molecular genetics centres with the aim of providing equal access to genetic tests for all cancer patients nationwide. The real bottleneck may be data analysis, as it may well be impossible for even a skilled oncologist to translate a complex molecular profile into a correct clinical decision without the use of automatic tools.

The third problem discussed was not, strictly speaking, a scientific problem at all, but a logistical one: how to ensure access to personalised drugs. These drugs are exceptionally expensive to develop and, without the guarantee of a large patient population, they will remain so in the clinic. The recently introduced targeted medicines that are currently available are often priced above internationally accepted ‘cost-effectiveness thresholds’ and so out of reach of healthcare systems even in many developed countries. This situation is only worsened by rising patient expectations, fuelled by media reports of personalised ‘magic bullets’ for all cancers that are always just round the corner. Schilsky suggested a possible solution in the creation of what he called a ‘global access programme’ in which each country would maintain a national formulary of representative targeted cancer drugs. Patient data would be entered in a national registry, and validated test results would be used to select the most appropriate drug for each patient from those available. The outcomes would be then be entered in the registry and used in re-evaluating and optimising the guidelines. A system like this could offer benefits for patients, physicians, drug companies, healthcare providers, and regulators, who would all be active participants in the endeavour of improving treatment options.

Schilsky ended his presentation, and the conference, by stressing the importance of education for clinicians. There is already a deluge of cancer-related molecular data that are threatening to overwhelm oncologists, let alone general physicians. ASCO is investing heavily in clinician training and has developed the CancerLinQ system to harness all the available data into automated analysis tools. One early benefit of this data aggregation is likely to identify patients with exactly the right combination of characteristics for enrollment into complex clinical trials.

The sixth WIN symposium is scheduled to take place, also in Paris, on 23 and 24 June 2014. Judging from the quality of the science presented at the fifth, this conference should be very worthwhile.

